# Modelling *TERT* regulation across 19 different cancer types based on the MIPRIP 2.0 gene regulatory network approach

**DOI:** 10.1186/s12859-019-3323-2

**Published:** 2019-12-30

**Authors:** Alexandra M. Poos, Theresa Kordaß, Amol Kolte, Volker Ast, Marcus Oswald, Karsten Rippe, Rainer König

**Affiliations:** 1 Integrated Research and Treatment Center, Center for Sepsis Control and Care (CSCC), Jena University Hospital, Am Klinikum 1, 07747 Jena, Germany; 2 Division of Chromatin Networks, German Cancer Research Center (DKFZ) and Bioquant, Im Neuenheimer Feld 267, 69120 Heidelberg, Germany; 3 Faculty of Biosciences, Heidelberg University, Heidelberg, Germany; 4 Research Group GMP & T Cell Therapy, German Cancer Research Center (DKFZ), Im Neuenheimer Feld 280, 69120 Heidelberg, Germany

**Keywords:** Mixed integer linear programming, Gene regulatory networks, Transcriptional regulation, Telomere maintenance, Telomerase, Cancer

## Abstract

**Background:**

Reactivation of the telomerase reverse transcriptase gene *TERT* is a central feature for unlimited proliferation of the majority of cancers. However, the underlying regulatory processes are only partly understood.

**Results:**

We assembled regulator binding information from serveral sources to construct a generic human and mouse gene regulatory network. Advancing our “Mixed Integer linear Programming based Regulatory Interaction Predictor” (MIPRIP) approach, we identified the most common and cancer-type specific regulators of *TERT* across 19 different human cancers. The results were validated by using the well-known *TERT* regulation by the ETS1 transcription factor in a subset of melanomas with mutations in the *TERT* promoter.

Our improved MIPRIP2 R-package and the associated generic regulatory networks are freely available at https://github.com/KoenigLabNM/MIPRIP.

**Conclusion:**

MIPRIP 2.0 identified common as well as tumor type specific regulators of *TERT*. The software can be easily applied to transcriptome datasets to predict gene regulation for any gene and disease/condition under investigation.

## Background

Telomere repeats are lost at the 3′-end erosion during replication of linear chromosomes. If the telomeres become critically short, senescence or apoptosis is induced. This process can thus act as a barrier towards unlimited proliferation and tumorigenesis [1]. Cancer cells circumvent this by acquiring a telomere maintenance mechanism (TMM) [2]. Usually, they reactivate the reverse transcriptase telomerase extending the telomere repeats [3, 4]. Human telomerase consists of the catalytic subunit *TERT* and the template RNA *TERC* (or hTR) [5]. *TERC* is constitutively expressed while the *TERT* gene is silenced in adult somatic cells [6, 7]. Germ and stem cells [7] as well as most tumor cells [2] express *TERT* so that telomerase is assembled. The mechanism of *TERT* activation in cancer cells appears to be highly variable between different cancer entities and numerous transcription factors (TFs) have been reported to be involved in this process [8–10]. The core region of the human *TERT* promoter is located between 330 bp upstream and 228 bp downstream of the transcription start site. This region comprises several TF binding sites, including binding sites with GC and E-box motifs [9]. Previous studies showed that *TERT* promoter mutations can induce its expression in cancer cells. *TERT* promoter mutations occur most frequently in bladder cancer (59%), cancers of the central nervous system (43%), melanoma skin cancer (29%) and follicular cell-derived thyroid cancer (10%) [11].

Here, we performed an in silico pan-cancer analysis of *TERT* regulation by using an evolved version of the “Mixed Integer linear Programming based Regulatory Interaction Predictor” (MIPRIP, version 2.0) to predict TFs regulating the gene expression of *TERT*. The new version bases on MIPRIP (https://github.com/KoenigLabNM/MIPRIP) which was previously developed to identify regulatory interactions that best explain the discrepancy of telomerase transcript levels in *Saccharomyces cerevisiae* between yeast deletion strains with shorter telomeres and strains with wild-type telomere length. In *S. cerevisiae* we uncovered novel regulators of telomerase expression, several of which affect histone levels or modifications [12]. A variety of other approaches have been developed which integrate regulatory information into a unified model of a gene regulatory network (GRN). Some of these approaches infer TF acitvity by linear regression employing gene expression profiles, a pre-defined network of TFs and their target genes [13–15], probabilistic models [16] or a reverse engineering approach that identifies regulator to target gene interactions from pairwise mutual information of their gene expression pofiles [17].

The activity of TFs frequently depends only partially on the gene expression of the TF itself but is rather modulated by post-translational modifications and protein stability. Hence, we and others inferred the activity of a TF from the expression of its potential target genes [13, 18, 19]. In the present study, we have optimized our MIPRIP software and applied it to gene expression profiles of 19 different cancer types from The Cancer Genome Atlas (TCGA) to identify TFs regulating the *TERT* gene.

## Results

### Transcription factor binding information and network construction

We constructed a generic human regulatory network based on seven different repositories, mainly containing experimental validated binding information from chromatin immunoprecipitation (ChIP) based assays. In total, the generic network comprises 618,537 interactions of 1160 regulators and 31,915 target genes. For *TERT*, we identified 75 putative regulators (Additional file 1: Table S1) that originated mainly from the manually curated database MetaCore™ (60 out of 75). Our list of *TERT* regulators compares well to the *TERT* regulators described in the review by Ramlee et al. [9]. Thirty from our assembly of 75 regulators were also described by Ramlee et al. (*P* = 6.01E-23, Fisher’s Exact Test) and except of CTCF (Encode) all were listed in MetaCore™. Additionally, we assembled a generic gene regulatory network for mouse containing 93,140 interactions of 976 TFs and 15,728 target genes from three different databases. To focus on more reliable potential interactions, entries were selected based on the presumed database reliability and co-occurrences.

### Three different modes of a MIPRIP 2.0 analysis

**Fig. 1:**
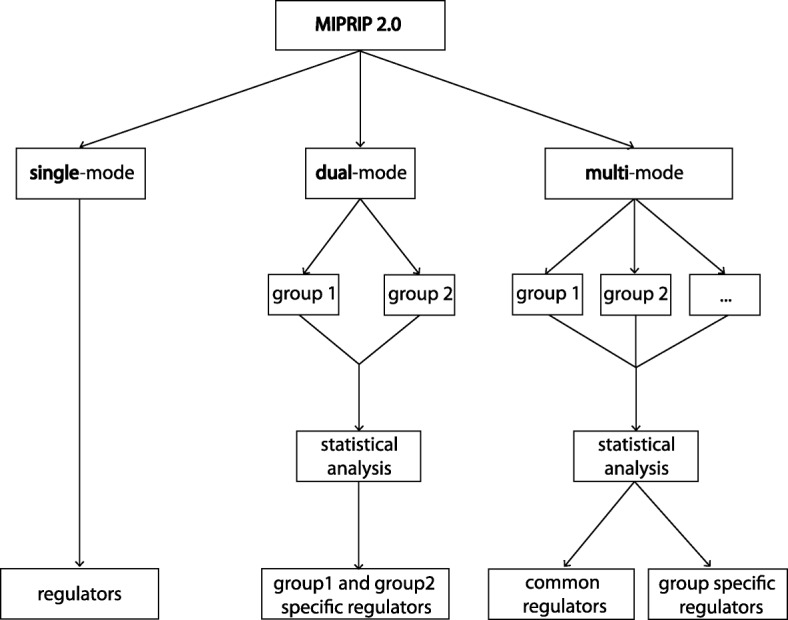
Schematic overview of the workflow. Three different modes are available in MIPRIP 2.0. The single-mode can be used to predict the most relevant regulators of the gene of interest based on a single entity of the disease or condition. The dual-mode compares the regulator predictions of a gene of interest between two different diseases or conditions (e.g. treatment versus control). The multi-mode can be used for more than two diseases or conditions to identify the most common and condition specific regulators of the gene of interest

MIPRIP 2.0 can be used to (i) predict the most important regulators of one group of samples (single-mode), (ii) identify significant regulators being different between two groups of samples (e.g. disease vs. control) (dual-mode) and (iii) can be applied to more than two groups (multi-mode). The newly developed multi-mode implementation is embedded in a statistical analysis pipeline and can be applied to more than two datasets or conditions to identify common but also condition-specific regulators (Fig. 1). Here, we applied the multi-mode MIPRIP 2.0 version to study the regulation of *TERT* across 19 different cancer types (described in the next section) and employed the dual-mode to compare the regulation of melanoma samples with and without *TERT* promoter mutation.

### Applying MIPRIP 2.0 to identify regulators of *TERT* across different cancers

**Fig. 2:**
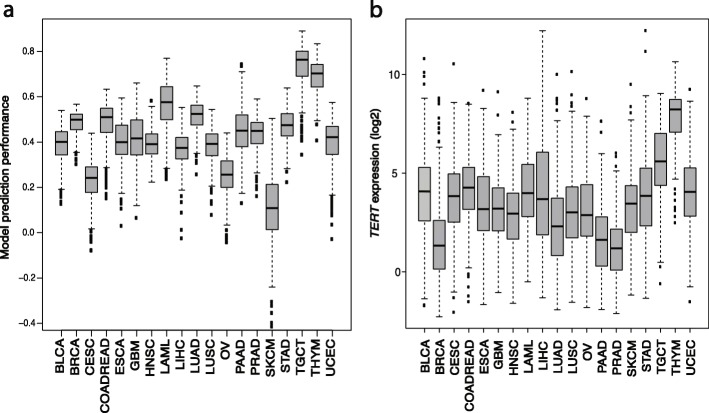
*TERT* expression and prediction performance for the investigated different cancer types. Boxplots for each cancer type of (**a**) the correlation between predicted and experimental gene expression over all models, and (**b**) *TERT* expression in each sample

We selected 19 different cancer types from TCGA (Additional file 1: Table S2) for which more than 100 primary tumor samples were available. For each cancer type, we set up a regulatory model for *TERT* by using a 10-times three-fold cross-validation. We calculated different models by restricting the numbers of maximal regulators from 1 up to 10 resulting in 300 models per cancer type. The performance of the models was estimated by the correlation between the predicted and the measured gene expression value (of *TERT* in the expression data). For most of the cancer types, the correlation was *r* = 0.4 or better (Fig. 2a). For cervical (CESC), ovary (OV) and melanoma skin (SKCM) cancer the performance was distinctively lower. The highest correlation was found for testicular germ cell cancer (TGCT) (*r* = 0.75) and thymoma (THYM) (*r* = 0.7), which also showed the highest *TERT* expression over all cancer types (Fig. 2b). The lowest *TERT* expression was found in breast (BRCA), pancreas (PAAD) and prostate (PRAD) cancer. The expression of *TERT* in melanoma skin cancer was comparable to most of the other cancer types, but the performance of the models was the worst (*r* = 0.1) (Fig. 2a). The performance could be increased by splitting up the melanoma skin cancer dataset into samples with and without *TERT* promoter mutation (see next section). As common regulators of *TERT* across all cancer types, we identified nine regulators: the two paired box proteins PAX5 and PAX8, the E2F factors 2 and 4, AR, BATF, SMARCB1, TAF1 and MXI1 (Table 1, all identified TFs are listed in Additional file 1: Table S3). To validate our results in silico, we queried Pubmed articles for the identified regulators. We found 21 out of 1002 *TERT* articles for our identified regulators which was a significant enrichment for our hits (*p* = 0.006, Additional file 1: Tables S4 and S5). When we performed the same Pubmed query with 9 randomly selected non-*TERT* regulators (*n* = 10), the enrichment was not significant. We performed an additional resampling test. To show that the prediction of *TERT* expression from our models is better than expected by a set of TFs selected by random chance, we randomly selected non-*TERT* TFs used by our model to predict the expression of *TERT* in the melanoma samples with a *TERT* promoter mutation. This was done 50 times. Averaging the results, the performance of the models was significantly better for the putative *TERT* TFs from the generic regulatory network (*r* = 0.29) compared to random non-*TERT* TFs (*r* = 0.18) *(p* = 3.744 E-15, T-Test). In summary, we applied MIPRIP 2.0 in the multi-mode and found nine TFs common to all investigated cancers predicted to regulate *TERT* expression and validated by screening the literature for co-occurrences.

**Table 1:** Predicted *TERT* regulators common to all 19 cancer types

TF	E-value
E2F4	0
AR	1.00 E-04
PAX5	4.00 E-04
E2F2	6.00 E-04
BATF	3.20 E-03
PAX8	6.30 E-03
SMARCB1	1.38 E-02
MXI1	1.87 E-02
TAF1	2.12 E-02

### Applying the dual-mode MIPRIP analysis to melanoma skin cancer

Melanoma skin cancer was the first cancer type for which a high frequency of *TERT* promoter mutations was discovered, mainly in two hotspot C > T mutations at position 124 bp and 146 bp upstream of the translational start codon [20, 21]. In the melanoma data we investigated, the *TERT* promoter mutation status was available for 115 samples. As described in the previous section, we obtained the lowest performance of our regulatory models for melanoma samples. Considering this and the high rate of *TERT* promoter mutations we divided the dataset into samples with and without *TERT* promoter mutation objecting to improve our predictions. We applied the MIPRIP 2.0 dual-mode to the separated datasets. Indeed, the performance could be increased to a correlation of *r* = 0.29. Employing this approach resulted in a list of 12 and 17 TFs which were significantly more often used in the models for the samples with and without *TERT* promoter mutation, respectively. AR, E2F1, JUND and ETS1 were the most significant regulators in the samples with *TERT* promoter mutation, while HMGA2, HIF1, RUNX2 and TAL1 were most significant in the samples without *TERT* promoter mutation (Table 2). To validate that ETS1 is a key regulator in the samples with *TERT* promoter mutation, we investigated published microarray data from experiments in which ETS1 was knocked down in melanoma cells with *TERT* promoter mutation [22]. Indeed, *TERT* expression was lower in the ETS1 knockdown sample compared to controls (fold change: 0.82). From the 54,675 affy probe-ids only 8688 probe-ids (15.9%) had a lower or equal fold change than *TERT* (see Additional file 1: Figure S1) in the knockdown samples evidencing the activating effect of ETS1 on *TERT* expression.

**Table 2:** *TERT* regulators of melanoma samples with (mut) and without (wt) *TERT* promoter mutation

Regulators in mut	*P*-value*	Regulators in wt	*P*-value**
AR	3.97 E-37	HMGA2	1.05 E-16
E2F1	3.00 E-29	HIF.1	1.03 E-15
JUND	2.86 E-25	RUNX2	2.88 E-12
SMARCB1	1.85 E-15	TAL1	1.54 E-09
ETS1	4.46 E-13	ESR2	3.92 E-09
SIN3AK20	1.42 E-06	AP-2	3.28 E-06
REST	3.64 E-06	MITF	2.59 E-05
MAZ	7.85 E-06	WT1	2.80 E-05
E2F2	9.20 E-05	SMAD3	5.76 E-05
TAF1	1.36 E-04	TFAP2D	2.78 E-04
BCL11A	4.31 E-04	PAX8	6.04 E-04
MYB	4.73 E-04	GRHL2	7.16 E-04
		TP53	1.21 E-03
		TCF7	1.68 E-03
		MZF1	3.44 E-03
		TFAP2C	3.68 E-03
		NR2F2	7.96 E-03

*indicates if a regulator was significantly more often selected in the samples with *TERT* promoter mutation compared to the wildtype

**indicates if a regulator was significantly more often selected in the wildtype compared to the *TERT* promoter mutated samples

To estimate the robustness of the choice for the upper limit of 10 potential regulators in our MIPRIP analysis, we investigated models with other limits (1 up to 50 regulators) and tested them using the melanoma skin cancer samples with a *TERT* promoter mutation. We found that models with a variety of different smaller numbers of candidate regulators led to comparable results (Additional file 1: Table S6). In all tested models between 1 and 20 regulators (1–5, 1–10, 5–10, 5–15, 1–20 or 5–20 regulators) AR was selected most often, followed by ETS1, JUND and E2F1. The performance of the models of 1 to 50 regulators is shown in Additional file 1: Figure S2. The figure shows that models with equal or less than 10 regulators yielded the best performance suggesting that too many regulators in the model can lead to overfitting. For models with more regulators, the performance reached a plateau and decreased. Therefore, models with 1 to 10 regulators suited well to predict the gene expression of *TERT* and this range was used for all analyses in the study.

In summary, splitting up the melanoma dataset into two pre-defined cancer subgroups with and without the *TERT* promoter mutations led to more reliable modelling results (*r* = 0.29). The dual-mode of MIPRIP 2.0 suited well to identify this specific regulation.

### Comparison with ISMARA

We compared our results with results from the well-established tool ISMARA [13]. Similar to MIPRIP, ISMARA identifies the activity of regulators based on their target genes [13]. In contrast to MIPRIP, target genes are inferred from motif binding information, and not directly from ChIP experiments. In ISMARA, TF activities are calculated for each sample alone and then averaged over the samples of each group (*TERT* promoter mutated versus wildtype). ISMARA identified 20 TFs for the *TERT* promoter (Additional file 1: Table S7), but only SIN3A, MAZ and WT1 overlapped with the MIPRIP 2.0 results. It is known that a *TERT* promoter mutation leads to a further binding site of TFs of the ETS family [20, 21]. From the ETS family of TFs, ISMARA predicted GABPA, ELF2 and ELF5 with very low significance, while MIPRIP identified ETS1 as a highly significant regulator of *TERT* in the melanoma samples with a *TERT* promoter mutation. In summary, the overlap between MIPRIP and ISMARA was low, particularly the highly significant hit of ETS1 by MIPRIP was in high agreement with the literature.

### Availability and implementation

MIPRIP 2.0 is implemented as a software package in R [23]. It is freely available on github [24]. MIPRIP 2.0 is platform independent and runs on R version 3.5.1 together with Gurobi version 8.0.1 and the CRAN R package slam.

## Discussion

In the present study we have advanced our software package “Mixed Integer linear Programming based Regulatory Interaction Predictor” (MIPRIP) for application to human and mouse cells. For this, we selected known regulator binding information to construct a generic network linking TFs to their potential target genes. The interactions between TFs and their targets organize as a scale-free network comprising hubs as central regulators [25]. TFs with the highest number of target genes in our generic human regulatory network were CTCF (*n* = 16,483), POLR2A (*n* = 16,076), TAF1 (*n* = 13,956), MYC (*n* = 13,692) and YY1 (*n* = 13,101), while more than half of the regulators had less than 25 target genes (Additional file 1: Figure S3). This might reflect the role of the former TFs as master regulators that recruit chromatin modifying co-factors and remodel the chromatin structure, as known for MYC [26], or mediate structural interactions between enhancers and promoters as it has been reported for CTCF and YY1 [27]. The edges (TF - target gene interactions) in the generic regulatory network were weighted based on the presumed database reliability, published earlier by us basing on a smaller set of databases [19]. Still, choosing the weights for these databases to optimality could be better worked out by setting a well-defined gold-standard data set, which e.g. could be derived from a set of well-known genes for which the regulation is well-understood and calibrating the weights against several reference expression data sets. We were interested how our method performs using the weighted edge scores compared to just the binary information. Instead of the edge scores, we applied a binary generic regulatory network in which entries equaled to one if the edge score was non-zero and otherwise zero. We applied this to data of the melanoma skin cancer samples with *TERT* promoter mutation. We again got ETS1, AR, JUND and E2F2 as regulators to be selected most often by the models showing that the results were quite comparable. However, the mean performance using the binary network was lower (*r* = 0.24) compared to the weighted generic regulatory network (*r* = 0.3) showing the improved performance when using the weighted edge scores.

The MIPRIP 2.0 framework with its new multi-mode was applied to dissect the regulation of the telomerase protein subunit *TERT* across 19 different cancer types, yielding nine TFs being common to *TERT* regulation across all cancer types. These identified regulators showed a significant enrichment in Pubmed entries for *TERT* in contrast to randomly selected TF combinations*.* Five TFs (PAX5, PAX8, AR, E2F2 and E2F4) have been described previously as *TERT* regulators. It has been reported that PAX5 has two and PAX8 four binding sites at the *TERT* transcription start site inducing activation of *TERT* transcription. Their function in telomerase regulation has been validated [28, 29]. The androgen receptor (AR) belongs to the class of nuclear receptors and is a repressor of *TERT* expression [30]. Bilsland et al. constructed a dynamic Boolean model to study *TERT* regulation in ovarian cancer cells. They identified MYC as an important player in *TERT* activation. In their model, loss of MYC led to suppression of *TERT*, which was substantially rescued only by a co-suppression of AR. Interestingly, in their model, *TERT* expression was well rescued by a gain of function of ETS [31]. This compares to its gain of function in the melanoma tumors with the *TERT* promoter mutation we studied. The E2F2 and E2F4 factors bind to the E2 recognition motif and are involved in cell cycle processes, DNA damage response [32] and regulate *TERT* transcription in human B-cell lymphoma [33, 34]. In addition to these five TFs, we identified BATF, SMARCB1, TAF1 and MXI1 as novel *TERT* regulators across cancer entities that to our knowledge, so far, have not been described in the literature as *TERT* regulators. Accordingly, we suggest these as potential candidates for future investigations on the mechanism of *TERT* reactivation in cancer cells.

The best performance of the MIPRIP 2.0 multi-mode analysis was observed for thymoma and testicular germ cell cancer, which showed also the highest *TERT* expression. The worst performance was observed for melanoma skin cancer, even though *TERT* expression was not particularly low. As described in the literature, cutaneous melanoma skin cancer patients have a high rate of *TERT* promoter mutations, being responsible for an upregulation of *TERT* by enabling a further binding site of TFs from the ETS family [20, 21]. Using MIPRIP 2.0 in the dual-mode after dividing the melanoma dataset into cancer samples with and without *TERT* promoter mutation improved the results considerably. We identified ETS1 as a highly significant regulator for *TERT* in tumors with *TERT* promoter mutation. To further validate this finding we analyzed publicly available expression data of an ETS1 siRNA knockdown experiment in a melanoma cell line with *TERT* promoter mutation and found a downregulation of *TERT* compared to controls. In line with this, ETS binding together with the activation of the non-canonical NFκB signaling pathway through the co-activator p52 enhances the promoter activity of *TERT* [35]. Furthermore, it was shown elsewhere that *TERT* promoter mutations can lead to a two- to four-fold higher *TERT* promoter activity in melanoma cells [20, 21]. We observed such an overexpression also in the analyzed melanoma dataset (*p*-value = 5.33 E-03).

Besides ETS1, we predicted AR, E2F1 and JUND as the most significant regulators in melanoma patients with a *TERT* promoter mutation. AR and E2F were also predicted as common *TERT* regulators in our multi-mode MIPRIP analysis. A recent study showed that an inhibition of E2F1 leads to increased cell death in melanoma cells, even if they are resistant to BRAF-inhibitors [36]. These results indicate that E2F1 is an interesting therapeutic target for melanoma. According to our predictions, E2F1 regulates samples with a *TERT* promoter mutation. As E2F1 is a *TERT* repressor [32], an inhibition of E2F1 may be more efficient in samples without *TERT* promoter mutation.

For melanoma samples without *TERT* promoter mutation, we predicted HMGA2, HIF1, RUNX2 and TAL1 as the most significant regulators. HMGA2 is a member of the high-mobility group of AT-hook proteins, which are expressed during embryonic development [37] as well as in different tumors (e.g. squamous cell carcinoma and malignant melanoma [38]). While only a few samples showed a *TERT* promoter mutation [38], it is still unclear if there is an association between HMGA2 expression and *TERT* promoter mutations. According to our predictions, we suggest that *TERT* regulation by HMGA2 and *TERT* promoter mutations are mutually exclusive, which has to be validated in future experiments.

In our case study, we observed that splitting up the datasets into subtypes led to an increased performance of the regulatory models and was necessary to break down the relevant regulatory processes. Melanoma patients with *TERT* promoter mutation show decreased survival rates [39]. Hence, identifying subtype specific regulatory mechanisms may support risk stratification by employing the identified regulators as biomarkers. In addition, such predictions may pave the way for a personalized therapy by developing drugs specifically interfering with the detected TFs.

Using the specific application of known ETS binding site in the *TERT* promoter of melanoma samples with a *TERT* promoter mutation as a case study, we compared the results from MIPRIP 2.0 with ISMARA [13]. The overlap between our results and ISMARA was very low. Particularly, ISMARA did not identify ETS1 as a regulator for *TERT* in samples with a *TERT* promoter mutation. GABPA, which is another member of the ETS-family, was predicted by ISMARA, but with rather low significance. It was shown elsewhere that GABPA can bind only to the *TERT* promoter mutation at site C228T, but not at C250T [40]. However, only one third of the mutated samples had the mutation at C228T, while two-third showed a C250T mutation [41]. In conclusion, comparing these two modeling approaches suggest that, compared to purely motif based methods like ISMARA, gene regulatory models basing also on experimental binding data like MIPRIP may easier detect a regulatory switch caused by genome mutations in TF – promoter binding regions. Still, for the future, further analysis with more case study examples is necessary giving more evidence for this.

The advancement of MIPRIP 2.0 compared to the previous version are the following: (1) MIPRIP 2.0 allows using the information of weighted edges while MIPRIP 1.0 could deal with the yeast regulatory network which based only on binary interaction values (binding of a regulator to the target gene was indicated by 1, otherwise 0). (2) A further advantage of MIPRIP 2.0 is the larger application variability by implementing three different modi (single-, dual- and multi-mode). Particularly, the multi-mode allows now also the comparative analysis of more than two datasets. Besides these advancements, MIPRIP 2.0 allows to extend the model by including information about gene copy number, DNA methylation, miRNA expression and binding, or additional variables e.g. related to further epigenetic regulation.

## Conclusions

We introduced MIPRIP 2.0 and applied it to predict *TERT* regulators in a pan-cancer analysis. Some of the common TFs identified, like PAX5, PAX8, AR, E2F2 and E2F4 have been previously described as *TERT* regulators. Others, like BATF, SMARCB1, TAF1 and MXI1, are novel. It will be exciting to test experimentally whether they are linked to a TMM phenotype. Furthermore, the predicted *TERT* regulators were compared in melanoma samples with wildtype versus mutated *TERT* promoters. We validated that a change of TF targets, in this case for TFs from the ETS family, was captured by MIPRIP 2.0. The software package is available on github [24] together with the generic human or mouse regulatory network and example datasets. It can be applied to a large variety of datasets to investigate the role of TF mediated gene regulation of a gene of interest in the context of diseases or other varying conditions.

## Methods

### Gene expression data

We downloaded publicly available transcriptome expression data (RNA-Seq) of all cancer types with more than 100 primary tumor samples from the TCGA Genome Data Analysis Center (GDAC) of the Broad Institute [42]. For these datasets the usage restriction has been lifted according to the TCGA publication guidelines from December 21, 2015 [43]. The pre-processed transcriptomic data with log2 transformed RSEM [44] normalized values were downloaded for 19 different cancer types listed in Additional file 1: Table S2. In each cancer type, genes with more than 25% missing entries and low varying genes (standard deviation ≤0.5) were filtered out. Furthermore, we performed a z-score transformation for each gene across each cancer dataset.

### Assembling transcription factor binding information into a generic human and mouse gene regulatory network

We assembled a comprehensive set of putative regulators for each gene by compiling TF binding information in human cells from seven different data repositories comprising (i) MetaCore™ [45] with annotated “direct”, “indirect” and “unspecific” interactions, (ii) the ChIP Enrichment Analysis (ChEA) database [46], (iii) ChIP data from the ENCODE project (http://www.genome.gov/Encode/), (iv) human ChIP-seq and ChIP-ChIP data from hmCHIP [47], (v) experimentally verified interactions from the Human Transcriptional Regulation Interactions database (HTRIdb) [48], (vi) ChIP-seq data for long non-coding RNA and microRNA genes from ChIPBase [49] and (vii) the method of Total Binding Affinity (TBA) [50]. TBA estimates the binding probability of a TF to the whole range of a gene’s promoter. Only TBA values with a score ≥ 1.5 were selected. All these repositories were used to compute the generic network of TFs and their target genes. Most interactions were extracted from Encode, followed by ChIPbase and hmChIP (Additional file 1: Figure S4A). Here, the highly reliable MetaCore™ interactions represent only 4% of all extracted TF-target gene interactions. An interaction between a TF *t* and a target gene *i* was considered if it was listed

(i) in MetaCore™ and labelled as direct, or listed in Encode (criteria 1),
(ii) in at least two out of MetaCore™ (labelled as indirect), ChEA, TBA (score ≥ 1.5) or HTRI (criteria 2), or
(iii) in ChIPBase and hmChIP (criteria 3).

Because of these criteria, several interactions were filtered out (Additional file 1: Figure S4B).

The different repositories were not equally incorporated due to the assumption, that some repositories were presumably more reliable than others. Because the interactions from MetaCore™ based on literature reports and were manually curated, MetaCore’s direct interactions (*MCdir*_*ti*_, activation, inhibition or unspecific) were weighted by a factor of 2, while MetaCore’s indirect interactions (*MCindir*_*ti*_, activation, inhibition or unspecific) were weighted by a factor of 1. Entries from *chea*_*ti*_*, htri*_*ti*_ and *tba*_*ti*_ were also weighted by a factor of 1, interactions from Encode (*enc*_*ti*_) by 0.5. A factor of 0.25 was used for interactions found in hmChIP (*hm*_*ti*_) and ChIPbase (*chip*_*ti*_). This led to the overall edge score *es*_*ti*:_

1 $$ {es}_{ti}:= 2\bullet {MCdir}_{ti}+0.5\bullet {enc}_{ti}+{a}_{ti}\bullet \left({MCindir}_{ti}+{chea}_{ti}+{htri}_{ti}+{tba}_{ti}\right)+0.25\bullet \left({hm}_{ti}\bullet {chip}_{ti}\right) $$

with

2 $$ {a}_{ti}:= \left\{\begin{array}{c}1\  if\ \left({MCindir}_{ti}+{chea}_{ti}+{htri}_{ti}+{tba}_{ti}\right)\ge 2\\ {}0\  else\end{array},\right. $$

and

3 $$ {MCdir}_{ti},\kern0.5em {enc}_{ti},{a}_{ti},{MCindir}_{ti},{chea}_{ti},{htri}_{ti},{tba}_{ti},{hm}_{ti},{chip}_{ti}\in \left\{0,1\right\} $$

In total the here presented generic network (version 1.0) comprised 618,537 non-zero entries for 1160 TFs and 31,915 target genes.

Similarly, a comprehensive set of putative regulators for each gene was assembled for mouse by compiling TF binding information from MetaCore™, ChEA and ENCODE containing TF binding information for mouse. Additionally, we added two more databases, ECRBase and TfactS. ECRBase is based on alignments of evolutionary conserved TF binding sites [51]. TfactS [52] contains interaction information inferred from the regulation of TFs from gene expression data of experimentally well-characterized target genes listed in TRED [53], TRRD [54], PAZAR [55] and NFIregulomeDB [56]. Interaction information of TF *t* and target gene *i* from MetaCore™ (MCdir_ti_) labelled as direct was weighted by 2. If an interaction was listed in two out of (a) MetaCore™ indirect (MCindir_ti_), (b) ChEA (chea_ti_) and (c) ECRbase (ecrbase_ti_), it was weighted by 1 (for each source). A listed mouse ENCODE entry (enc_ti_) was weighted by 0.5. The interactions of TfactS (tfacs_ti_) were considered to have weaker evidence and were weighted by 0.25. This led to the overall edge score *mes*_*ti*_ for mouse:

4 $$ {mes}_{ti}:= 2\bullet {MCdir}_{ti}+{a}_{ti}\bullet \left({MCindir}_{ti}+{chea}_{ti}+{ecrbase}_{ti}\right)+0.5\bullet {enc}_{ti}+0.25\bullet {tfacs}_{ti} $$

with

5 $$ {a}_{ti}:= \left\{\begin{array}{c}1\  if\ \left({MCindir}_{ti}+{chea}_{ti}+{ecrbase}_{ti}\right)\ge 2\\ {}0\  else\end{array}\right. $$

and

6 $$ {MCdir}_{ti},{MCindir}_{ti},{chea}_{ti},{ecrbase}_{ti},{enc}_{ti},{tfacs}_{ti},{a}_{ti}\in \left\{0,1\right\} $$

In total the generic mouse network (version 1.0) comprises 93,140 non-zero entries for 976 TFs and 15,728 target genes.

### Modeling *TERT* regulation

We optimized our previously developed “Mixed Integer linear Programming based Regulatory Interaction Predictor” (MIPRIP) software (https://github.com/KoenigLabNM/MIPRIP) [12]. MIPRIP 2.0 can be used for one set of samples (single-mode), can be applied to compare the regulatory processes between two sets of samples (dual-mode), and for multiple datasets, to identify the most common and condition specific regulators (multi-mode) (Fig. 1). The basic idea of MIPRIP is to identify the most relevant regulators of a particular target gene by predicting the target gene’s expression using a linear model in which the covariates are all potential regulators putatively binding to its promoter. In this study, MIPRIP was applied to predict the regulators of the *TERT* gene. The gene expression value ${\overset{\sim }{g}}_{TERT,\kern0.5em k}$ of *TERT* was predicted for each sample by the following model:

7 $$ {\overset{\sim }{g}}_{TERT,k}={\beta}_0+\sum \limits_{t=1}^T{\beta}_t\bullet {es}_{t, TERT}\bullet {act}_{tk} $$

where *β*_*0*_ was an additive offset, *T* the number of all regulators for which *TERT* promoter binding information was available, *β*_*t*_ was the optimization parameter for regulator *t*, *es*_*ti*_ was the edge score between regulator *t* and its putative target gene *i* and *act*_*tk*_ the activity of regulator *t* in sample *k*. If gene *i* was reported to be a target of regulator *t*, the edge weight was higher than 0. Instead of using the gene expression value of a regulator, we calculated an activity value *act*_*tk*_ for each regulator and each sample based on the expression of all its putative target genes *g*_*ik*_ by

8 $$ {act}_{tk}=\frac{\sum_{i=1}^n{es}_{ti}\bullet {g}_{ik}}{\sum_{i=1}^n{es}_{ti}} $$

The activity is the cumulative effect of a regulator on all its target genes, normalized by the sum of all target genes. To calculate the activity value, we excluded the expression value of the gene of interest (*TERT*) itself. A linear regression was performed based on Mixed Integer Linear Programming (MILP). MILP has advantages over the lasso regression model, as in a MILP based regression, the error penalties are linear (L1 regression) and not quadratic which avoids over-emphasizing outliers. Furthermore, MILP enables using binary on-off switches for each beta coefficient to limit the number of beta coefficients [for details, see [19]]. All linear equations are optimized using the Gurobi optimizer [57] (version 6.0–7.01) to minimize the difference between the measured transcript level (from the gene expression matrix) *g*_*TERT*, *k*_ and the predicted gene expression ${\overset{\sim }{g}}_{TERT,k}$ value. This equals to minimizing the error terms *e*_*TERT*, *k*_ in

9 $$ \min {\sum}_{k=1}^l\left|{g}_{TERT,k}-{\overset{\sim }{g}}_{TERT,k}\right|=\sum \limits_{k=1}^l{e}_{TERT,k} $$

Because MILP cannot handle absolute values, the absolute values were transformed into two inequalities for each gene *i* and sample *k*,

10 $$ {g}_{TERT,k}-{\overset{\sim }{g}}_{TERT,k}-{e}_{TERT,k}\le 0 $$

11 $$ -{g}_{TERT,k}+{\overset{\sim }{g}}_{TERT,k}-{e}_{TERT,k}\le 0 $$

In general, non-trivial models can be constructed starting with only one regulator up to a maximum of m = *n-2* putative regulators, where *n* is the number of samples. In our example, the number of regulators m was not a critical parameter. The results for different ranges of regulators (m = 1–5, 1–10, 5–10, 1–20, 5–20 and 1–50) was comparable (listed in Additional file 1: Table S6, see results).

To constrain the number of regulators, a binary variable was introduced for each regulator *t* called *x*_*t*_ ∈ {0, 1}. If regulator *t* was selected by the model, *x*_*t*_ was equal to 1, and zero else. The maximal allowed sum of all binary *x*_*t*_ variables was constrained by the variable *limit*,

12 $$ {x}_1+{x}_2+\cdots +{x}_t\le limit;{x}_t\in \left\{0,1\right\} $$

*limit* ranged from 1 to 50 depending on the predefined maximal number of regulators in the model. Furthermore, a variable called ‘Big M’ was utilized to implement these binary decisions. This implied to define the bounds of variable *β*_*t*_ of regulator *t*. The bounds of each *β*_*t*_ were set to −1000 ≤ *β*_*t*_ ≤ 1000 by

13 $$ {\beta}_t-M\ {x}_t\le 0 $$

14 $$ {\beta}_t+M\ {x}_t\ge 0 $$

with M = 1000. To avoid overfitting, a 10-times threefold cross-validation was performed yielding 300 models for each dataset. The Pearson correlation coefficient was calculated for the measured and the predicted gene expression values from the models to estimate the prediction performance.

### Single-mode MIPRIP 2.0 analysis

The single-mode analysis was developed to predict a list of regulators best explaining the gene expression profile of the target gene of interest (*TERT*) for all samples of a dataset for a single condition or disease. The single mode has no additional statistical analysis beyond the linear modelling. The results are simply the frequency of regulators over all cross-validation runs, prioritized by their usages.

### Dual-mode MIPRIP 2.0 analysis

As a case study, we applied the dual-mode analysis to the skin cutaneous melanoma (SKCM) dataset. The SKCM dataset was divided into two subgroups based on the *TERT* promoter mutation (based on the analysis of [41]). In total, the status of the *TERT* promoter was available for 115 samples (primary and metastatic samples). One subgroup (*n* = 74) comprised samples with a *TERT* promoter mutation, the other subgroup comprised samples with the according wildtype of the *TERT* promoter (*n* = 41). With these two subgroups we performed a dual-mode analysis by calculating the linear models using the same parameters as described above. Significant regulators between the two subgroups were determined by a two-sided Fisher’s Exact Test, testing an enrichment of a TF to be in a model of the first or the second condition based on their distribution in the different models, followed by multiple testing correction using the Benjamini-Hochberg method [58]. The stringency cutoff was set to *P* = 0.01.

### Multi-mode MIPRIP 2.0 analysis

The multi-mode analysis was developed to predict (1) a list of regulators best explaining the gene expression of the gene of interest (*TERT*) across all conditions (in our case tumor types), and (2) for each specific condition, in contrast to all other conditions. We prioritized the regulators as follows. For each condition, we listed how often each regulator was selected by the optimizer resulting in a count table. With these distributions we performed a one-sided Wilcoxon Test for each regulator in the list to identify regulators which were selected significantly more often in one of the conditions compared to all other conditions yielding the condition specific regulators. Significance values (*p*-values) were corrected for multiple testing [58]. To identify the most common *TERT* regulators within all conditions (of all 19 cancer types), a rank product test was performed based on the ranks from the counts of each condition. A permutation-based estimation was used to determine if the rank product value was higher than an observed value from a random distribution. We then counted how often the rank product values in the permutations were below or equal to the observed value, which led to an averaged expected value (E-value) [59].

### Systematic literature query

To validate the identified common *TERT* regulators from the multi-mode MIPRIP 2.0 analysis with data from the literature, a Pubmed [60] search was performed. For this purpose, all nine identified regulator gene symbols (from Table 1) were queried together with the terms “TERT” and “telomerase”, “human” and “regulation”. The query was “(E2F4 OR AR OR PAX5 OR E2F2 OR BATF OR PAX8 OR SMARCB1 OR MXI1 OR TAF1) AND TERT AND telomerase AND human AND regulation”. The received number of articles was compared to the number of articles from a query without the identified regulators. The query for this was “TERT AND telomerase AND human AND regulation”. For the background, the same two queries were performed without the “TERT” gene symbol. Using the results of these queries, a Fisher’s Exact Test was performed to test if the nine identified regulators were found significantly more often together with *TERT* than without *TERT*.

### TF perturbation experiments

We investigated *TERT* expression upon ETS1 knockdown for validating our result of the dual-mode case study. Previously, Wang et al. performed siRNA mediated knockdowns of 45 TFs and signaling molecules in the melanoma cell line A375. The gene expression of cells with the knockdown (1 sample per knockdown), untreated (3 replicates) and siRNA control treated (3 replicates) cells was profiled using microarrays (Affymetrix GeneChip Human Genome U133 Plus 2.0) 48 h after transfection [22]. RMA-normalized expression data of these perturbation experiments was downloaded from Gene Expression Omnibus (GSE31534). Affy probe-ids were mapped to gene symbols using BioMart [61] and expression values of multiple affy probe-ids for the same gene were averaged. A fold change was calculated for *TERT* upon ETS1 knockdown compared to the controls.

### Comparison of MIPRIP 2.0 with ISMARA

We compared our MIPRIP 2.0 results with the results from the “Integrated Motif Activity Response Analysis” (ISMARA) tool. ISMARA predicts regulatory interactions between the TFs and the target genes based on TF binding motifs [13]. For the SKCM data from TCGA only preprocessed data was available. Hence, ISMARA could not be used via the web portal. Therefore, the ISMARA analysis was performed by the developers of ISMARA using FPKM values (downloaded from the GDC portal [62], June 2018) and default settings. For comparison, MIPRIP models were also constructed using these FPKM values (log2- and z-transformed) instead of the RSEM normalized counts. For both datasets, the regulators AR, JUND, E2F1, E2F2 and ETS1 were selected most often (Additional file 1: Table S6, column “FPKM”).

## Supplementary information

**Additional file 1: Table S1.** List of transcription factors putatively regulating *TERT*, based on the generic human gene regulatory network. **Table S2.** Selected cancers from The Cancer Genome Atlas. **Table S3.** Specific *TERT* regulators of each cancer type. **Table S4.** Number of Pubmed hits for the predicted common *TERT* regulators. **Table S5.** Confusion matrix for the Pubmed query. **Table S6.** Estimating the best model size. **Table S7.** *TERT* regulators predicted by ISMARA. **Figure S1.** Histogram of the fold changes of the TF knockouts in the melanoma cell line A375 compared to controls. **Figure S2.** Performance of the melanoma skin cancer models with 1 to 50 regulators. **Figure S3.** Number of target genes identified for the 1160 TFs. **Figure S4.** TF-target gene interactions.

## Data Availability

The R-package ‘MIPRIP2’ and the generic human/mouse regulatory networks can be downloaded from https://github.com/KoenigLabNM/MIPRIP. All datasets analyzed in this study are available at the GDAC (http://gdac.broadinstitute.org/, Firehose stddata__2016_01_28 run) or the GDC (https://portal.gdc.cancer.gov/, TCGA-PRAD, June 2018) portal.

## Abbreviations

AR: Androgen receptor
BRCA: Breast cancer
CESC: Cervical cancer
ChEA: ChIP Enrichment Analysis
ChIP: Chromatin immunoprecipitation
EMSA: Electrophoretic shift assay
es: edge score
ETS: E-twenty six
E-value: Expected value
FPKM: Fragments per kilobase of exon model per million reads mapped
GDAC: Genome Data Analysis Center
GRN: Gene regulatory network
HTRIdb: Human Transcriptional Regulation Interactions database
ISMARA: Integrated Motif Activity Response Analysis
MILP: Mixed Integer Linear Programming
MIPRIP: Mixed Integer linear Programming based Regulatory Interaction Predictor
OV: Ovarian serous cystadenocarcinoma
PAAD: Pancreatic ductal adenocarcinoma
PRAD: Prostate adenocarcinoma
RSEM: Accurate transcript quantification from RNA-Seq data with or without a reference genome
SKCM: Skin cutaneous melanoma
TBA: Total binding affinity
TCGA: The Cancer Genome Atlas
TERT: Telomerase reverse transcriptase
TF: Transcription factor
TGCT: Testicular germ cell cancer
THYM: Thymoma
TMM: Telomere maintenance mechanism
