# Supplementary material for: Modelling TERT regulation across 19 different cancer types based on the MIPRIP 2.0 gene regulatory network approach
Source: BMC Bioinformatics. 2019 Dec 30;20:737. doi: 10.1186/s12859-019-3323-2 (PMC6937852; doi:10.1186/s12859-019-3323-2)
Supplement: Supplementary file 1 — Additional file 1: Table S1. List of transcription factors putatively regulating TERT, based on the generic human gene regulatory network. Table S2. Selected cancers from The Cancer Genome Atlas. Table S3. Specific TERT regulators of each cancer type. Table S4. Number of Pubmed hits for the predicted common TERT regulators. Table S5. Confusion matrix for the Pubmed query. Table S6. Estimating the best model size. Table S7. TERT regulators predicted by ISMARA. Figure S1. Histogram of the fold changes of the TF knockouts in the melanoma cell line A375 compared to controls. Figure S2. Performance of the melanoma skin cancer models with 1 to 50 regulators. Figure S3. Number of target genes identified for the 1160 TFs. Figure S4. TF-target gene interactions. [file 12859_2019_3323_MOESM1_ESM.zip › Supplementary_material.pdf]

Modelling *TERT* regulation across 19 different cancer types based on the MIPRIP 2.0 gene regulatory network approach

Alexandra M. Poos[1,2,3], Theresa Kordaß[4,3], Amol Kolte[1], Volker Ast[1], Marcus Oswald[1], Karsten Rippe[2] and Rainer König[1]

[1] Integrated Research and Treatment Center, Center for Sepsis Control and Care (CSCC), Jena University Hospital, Am Klinikum 1, 07747 Jena, Germany
[2] Division of Chromatin Networks, German Cancer Research Center (DKFZ) and Bioquant, Im Neuenheimer Feld 267, 69120 Heidelberg, Germany
[3] Faculty of Biosciences, Heidelberg University, Heidelberg, Germany
[4] Research Group GMP & T Cell Therapy, German Cancer Research Center (DKFZ), Im Neuenheimer Feld 280, 69120 Heidelberg, Germany

Supplementary Material

Table S1: List of transcription factors putatively regulating *TERT*, based on the generic human gene regulatory network

TF	Edge strength score	TF	Edge strength score	TF	Edge strength score
**AP-2**	2.00	**HMGA2**	2.00	**PITX1**	2.00
**AR**	2.00	**HNRNPK**	2.00	**POLR2A**	0.50
**BATF**	0.25	**IKZF1**	2.00	**POU2F2**	0.25
**BCL11A**	0.25	**IRF1**	2.00	**RAD21**	0.50
**BHLHE40**	2.00	**JUND**	2.00	**RELA**	2.00
**CEBPA**	2.00	**KLF2**	2.00	**REST**	0.25
**CTCF**	0.50	**MAX**	2.50	**RUNX2**	2.00
**CTCFL**	2.00	**MAZ**	2.00	**SIN3A**	0.50
**E2F1**	2.00	**MEN1**	2.00	**SIN3AK20**	0.50
**E2F2**	2.00	**MITF**	2.00	**SMAD3**	2.00
**E2F4**	2.25	**MXD1**	2.00	**SMARCB1**	0.25
**E2F5**	2.00	**MXI1**	0.50	**SP3**	2.00
**E2F6**	2.25	**MYB**	2.00	**TAF1**	0.25
**EGR1**	2.50	**MYC**	3.75	**TAF9**	2.00
**EPAS1**	2.00	**MYCN**	2.00	**TAL1**	2.00
**ESR1**	2.00	**MZF1**	2.00	**TCF12**	0.25
**ESR2**	2.00	**NFAT5**	2.00	**TCF7**	2.00
**ETS1**	2.00	**NFATC2**	2.00	**TFAP2A**	2.00
**ETS2**	2.00	**NF.KB**	2.00	**TFAP2B**	2.00
**GLI1**	2.00	**NFKB1**	1.00	**TFAP2C**	2.00
**GLI2**	2.00	**NFKB.P50.P65**	2.00	**TFAP2D**	2.00
**GRHL2**	2.00	**NFX1**	2.00	**TP53**	2.00
**HEY1**	0.25	**NR2F2**	2.00	**TP73**	2.00
**HIF.1**	2.00	**PAX5**	2.00	**WT1**	2.00
**HIF1A**	2.00	**PAX8**	2.00	**ZBTB48**	2.00

Table S2: Selected cancers from The Cancer Genome Atlas

Cancer type	Number of tumor samples
Breast cancer (BRCA)	983
Cervical cancer (CESC)	300
Colorectal adenocarcinoma (COADREAD)	619
Cutaneous melanoma (SKCM)	103
Glioblastoma multiforme (GBM)	145
Head and neck squamous cell carcinoma (HNSC)	501
Liver hepatocellular carcinoma (LIHC)	342
Lung adenocarcinoma (LUAD)	491
Lung squamous cell carcinoma (LUSC)	489
Ovarian serous cystadenocarcinoma (OV)	294
Prostate adenocarcinoma (PRAD)	445
Stomach adenocarcinoma (STAD)	405
Urothelial bladder cancer (BLCA)	399
Uterine corpus endometrial carcinoma (UCEC)	532
Acute Myeloid Leukemia (LAML)	168
Testicular germ cell cancer (TGCT)	148
Esophageal cancer (ESCA)	178
Pancreatic ductal adenocarcinoma (PAAD)	145
Thymoma (THYM)	120

Table S3: Specific *TERT* regulators of each cancer type (see Excel-file)

Table S4: Number of Pubmed hits for the predicted common *TERT* regulators

Regulator	Pubmed hits with *TERT* (AND “telomerase” AND “human” AND “regulation”)	Pubmed hits without *TERT* (AND ”telomerase” AND “human” AND “regulation”)
**E2F4**	0	3
**AR**	15	35
**PAX5**	2	2
**E2F2**	1	2
**BATF**	2	2
**PAX8**	1	3
**SMARCB1**	0	0
**MXI1**	0	0
**TAF1**	0	0

Table S5: Confusion matrix for the Pubmed query

	Found with the query containing the nine predicted regulators	Found only with the query which did not contain the nine predicted regulators
**Query with TERT**	22	1,005
**Query w/o TERT**	18	2,052

Table S6: Estimating the best model size (see Excel-file)

Table S7: ***TERT*** **regulators predicted by ISMARA**

Regulator	Score
AHR_ARNT2	0.38
ARNT	0.73
CTCF_CTCFL	0.85
ELF2_GABPA_ELF5	0.01
ELK4_ETV5_ELK1_ELK3_ELF4	1.41
GMEB2	1.11
HES1	0.33
IKZF1	0.23
KLF16_SP2	1.48
MAZ_ZNF281_GTF2F1	0.41
MNT_HEY1_HEY2	1.05
MXI1_MYC_MYCN	3.40
MYF6	0.10
PLAGL1	1.37
RCOR1_MTA3	0.85
SIN3A_CHD1	0.84
SIX4	1.31
TCF3_MYOG	0.32
TCF12_ASCL2	1.05
WT1_MTF1_ZBTB7B	0.04

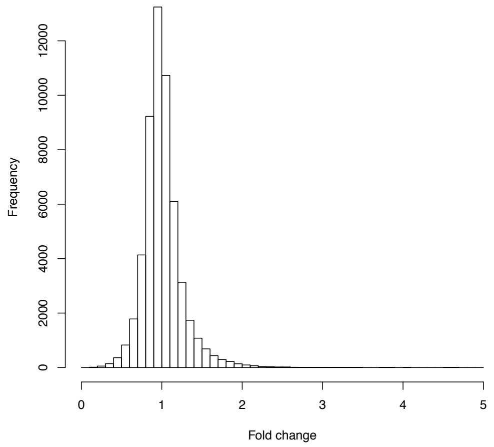

Figure S1. Histogram of the fold changes of the TF knockouts in the melanoma cell line A375 compared to controls

From the 54,675 affy probe-ids only 8,688 probe-ids (15.9 %) have a lower or equal fold change than *TERT.*

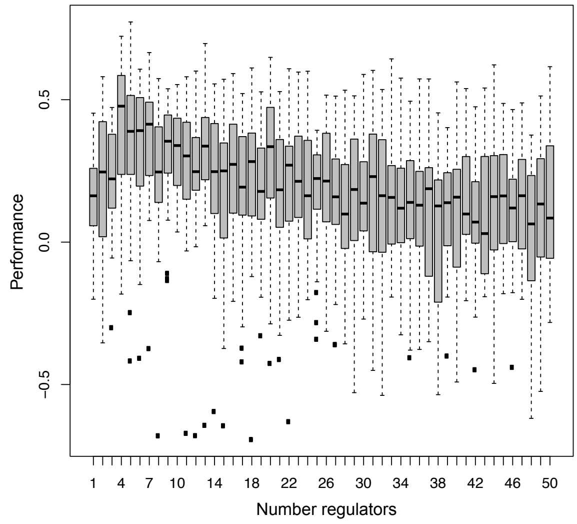

Figure S2. Performance of the melanoma skin cancer models with 1 to 50 regulators

Here, the performance of the models with 1-50 regulators is plotted over all cross-validation runs for the melanoma skin cancer samples with a *TERT* promoter mutation. Models with a smaller number of regulators showed a better performance than models with a high number of regulators.

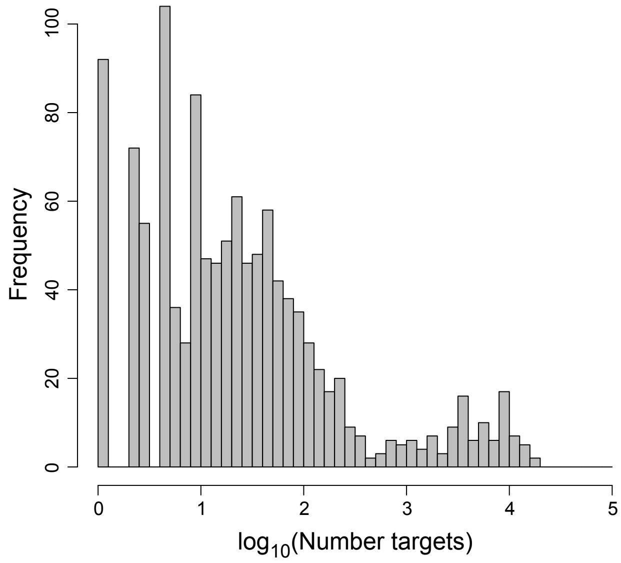

Figure S3. Number of target genes identified for the 1,160 TFs.

The histogram shows the scale-free structure of the generic human regulatory network. Some regulators had up to 17,000 target genes, while more than half of the regulators had less than 25 target genes.

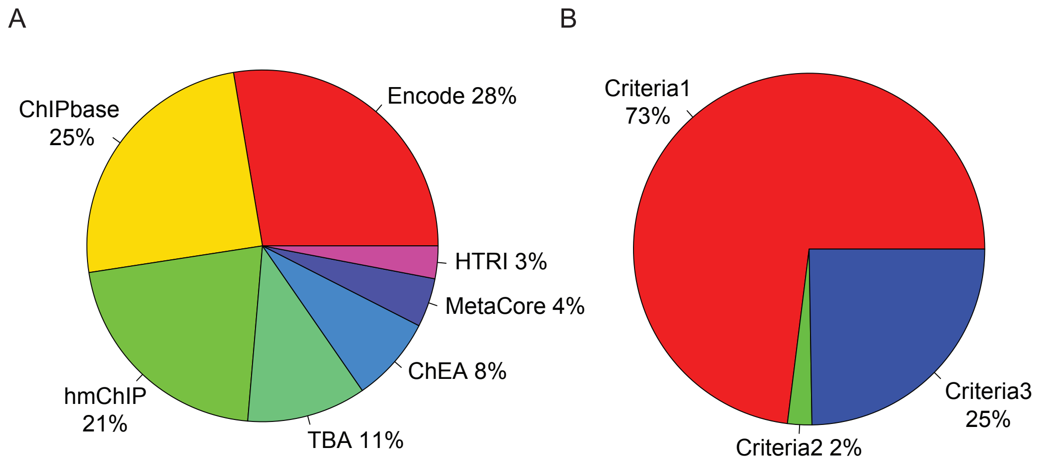

Figure S4. TF-target gene interactions.

(A) extracted from the 7 different sources and (B) fulfilling the defined criteria.
